# HDL cholesterol efflux capacity in rheumatoid arthritis patients: contributing factors and relationship with subclinical atherosclerosis

**DOI:** 10.1186/s13075-017-1311-3

**Published:** 2017-05-31

**Authors:** Beatriz Tejera-Segura, María Macía-Díaz, José David Machado, Antonia de Vera-González, Jose A. García-Dopico, José M. Olmos, José L. Hernández, Federico Díaz-González, Miguel A. González-Gay, Iván Ferraz-Amaro

**Affiliations:** 1 Division of Rheumatology, Hospital Universitario de Canarias, 38320 Santa Cruz de Tenerife, Spain; 2 Department of Pharmacology, Facultad de Medicina, Universidad de La Laguna, Tenerife, Spain; 3 Central Laboratory Division, Hospital Universitario de Canarias, Tenerife, Spain; 4 Division of Internal Medicine, Hospital Universitario Marqués de Valdecilla- Instituto de Investigación Marqués de Valdecilla (IDIVAL), Universidad de Cantabria, Santander, Spain; 5 Department of Internal Medicine, Facultad de Medicina, Universidad de La Laguna, La Laguna, Spain; 6 Division of Rheumatology, Hospital Universitario Marqués de Valdecilla, Universidad de Cantabria, Santander, Spain; 7 Epidemiology, Genetics and Atherosclerosis Research Group on Systemic Inflammatory Diseases, Hospital Universitario Marqués de Valdecilla, Instituto de Investigación Marqués de Valdecilla (IDIVAL), Santander, Spain; 8 Cardiovascular Pathophysiology and Genomics Research Unit, School of Physiology, Faculty of Health Sciences, University of the Witwatersrand, Johannesburg, South Africa

**Keywords:** Rheumatoid arthritis, Cholesterol efflux capacity, Carotid intima-media thickness, Cardiovascular disease

## Abstract

**Background:**

Lipid profiles appear to be altered in rheumatoid arthritis (RA) patients because of disease activity and inflammation. Cholesterol efflux capacity (CEC), which is the ability of high-density lipoprotein cholesterol to accept cholesterol from macrophages, has been linked not only to cardiovascular events in the general population but also to being impaired in patients with RA. The aim of this study was to establish whether CEC is related to subclinical carotid atherosclerosis in patients with RA.

**Methods:**

We conducted a cross-sectional study that encompassed 401 individuals, including 178 patients with RA and 223 sex-matched control subjects. CEC, using an in vitro assay, lipoprotein serum concentrations, and standard lipid profile, was assessed in patients and control subjects. Carotid intima-media thickness (CIMT) and carotid plaques were assessed in patients with RA. A multivariable analysis was performed to evaluate the relationship of CEC with RA-related data, lipid profile, and subclinical carotid atherosclerosis.

**Results:**

Mean (SD) CEC was not significantly different between patients with RA (18.9 ± 9.0%) and control subjects (16.9 ± 10.4%) (*p* = 0.11). Patients with RA with low (β coefficient −5.2 [−10.0 to 0.3]%, *p* = 0.039) and moderate disease activity (β coefficient −4.6 [−8.5 to 0.7]%, *p* = 0.020) were associated with lower levels of CEC than patients in remission. Although no association with CIMT was found, higher CEC was independently associated with a lower risk for the presence of carotid plaque in patients with RA (odds ratio 0.94 [95% CI 0.89–0.98], *p* = 0.015).

**Conclusions:**

CEC is independently associated with carotid plaque in patients with RA.

## Background

Patients with rheumatoid arthritis (RA) have higher rates of morbidity and mortality than the general population, which has been widely attributed to these patients’ increased risk of cardiovascular disease [1]. Besides a genetic component and the influence of traditional cardiovascular risk factors [2], chronic inflammation seems to play a pivotal role in the process of accelerated atherogenesis observed in RA [3]. Several studies support the notion that RA leads to a more atherogenic lipid profile, which correlates with disease activity and improves after treatment with antirheumatic medications [4]. Paradoxically, these lipid changes generally account for a decrease in total cholesterol and low-density lipoprotein (LDL) cholesterol [5, 6]. The exact mechanism that leads to this paradoxical lipid profile in patients with RA and the implications regarding cardiovascular risk are still being studied.

High-density lipoprotein (HDL) cholesterol efflux capacity (CEC), which is the ability of HDL to accept cholesterol from macrophages, is a key step in reverse cholesterol transport. It has been shown to be inversely associated, independent of HDL cholesterol levels, with both carotid intima-media thickness (CIMT) and the likelihood of angiographic coronary artery disease [7], as well as with a higher incidence of cardiovascular events, in population-based cohorts [8]. It is known that CEC is impaired in patients with RA and that this correlates with systemic inflammation and HDL’s antioxidant capacity [9, 10]. However, the implications of this impaired CEC in the development of subclinical atherosclerosis in patients with RA have not yet been studied.

The aim of this study was to analyze whether CEC is related to subclinical atherosclerosis as determined by the presence of carotid plaque or increased levels of CIMT in patients with RA. Secondarily, we aimed to describe the disease-contributing factors that are related to CEC as an expression of the abnormalities in the lipid profile associated with the disease. If CEC is related to subclinical atherosclerosis in patients with RA, this effect would shed light on the complex connections between inflammation, lipid abnormalities, and subclinical atherosclerosis in RA.

## Methods

### Study participants

This was a cross-sectional study that included 401 individuals, 223 patients with RA, and 178 sex-matched control subjects. All patients with RA were 18 years old or older and fulfilled the 2010 American College of Rheumatology/European League Against Rheumatism diagnostic criteria for RA [11]. They had been diagnosed by rheumatologists and were periodically followed at rheumatology outpatient clinics. For the purpose of inclusion in the present study, RA disease duration was required to be ≥1 year. Although anti-tumor necrosis factor-α (anti-TNF-α) treatment has been associated with changes in lipid profiles [12], patients with RA undergoing TNF-α antagonist or other biologic therapies were not excluded from the present study. The control group consisted of patients recruited from the Spanish Camargo Cohort Study [13, 14]. This cohort was set up between February 2006 and February 2011, and individuals included in this cohort have been followed ever since. The original aim of using this cohort was to evaluate the prevalence and incidence of metabolic bone diseases and mineral metabolism disorders. Control subjects included in the present study were sex-matched subjects without any known condition or drug treatment history that could influence lipids and who were not taking any lipid-lowering medications other than statins. None of the control subjects were receiving glucocorticoids. However, because prednisone is often used in the management of RA, patients taking prednisone or an equivalent dose ≤10 mg/day were not excluded. As previously mentioned, both patients and control subjects receiving statin treatment were allowed to participate in the study. Patients and control subjects were excluded if they had a history of myocardial infarction, angina, stroke, a glomerular filtration rate <60 ml/minute/1.73 m^2^, a history of cancer or any other chronic disease, or evidence of active infection. The study protocol was approved by the institutional review committees at Hospital Universitario de Canarias and Hospital Universitario Marqués de Valdecilla (both in Spain), and all subjects provided written informed consent.

### Data collection

The subjects, both control subjects and patients, completed a cardiovascular risk factor and medication use questionnaire and underwent a physical examination. Weight, height, body mass index, waist-to-hip ratio, and systolic and diastolic blood pressure (measured with the participant in a supine position) were assessed under standardized conditions. Information regarding smoking status (current smoker versus nonsmoker), diabetes, and hypertension was obtained from the questionnaire. Medical records were reviewed to ascertain specific diagnoses and medications. Dyslipidemia was defined as present if one of the following was measured: total cholesterol >200 mg/dl, triglycerides >150 mg/dl, HDL cholesterol <40 in men or <50 mg/dl in women, or LDL cholesterol >130 mg/dl. Disease activity in patients with RA was measured using the Disease Activity Score in 28 joints (DAS28) [15], the Clinical Disease Activity Index (CDAI) [16], and the Simplified Disease Activity Index [17]. Patients with RA were defined as being in clinical remission (DAS28 < 2.6) or having low (DAS28 in the range of 2.6–3.2), moderate (DAS28 > 3.2–5.1), or high (DAS28 > 5.1) disease activity as previously described [18].

### Lipids and cholesterol efflux assessments

Fasting serum samples were collected and frozen at −80 °C until analysis of circulating lipids. Cholesterol, triglycerides, and HDL cholesterol were measured using an enzymatic colorimetric assay (Roche Diagnostics, Indianapolis, IN, USA). Cholesterol levels ranged from 0.08 to 20.7 mmol/L (intra-assay coefficient of variation 0.3%); triglyceride levels ranged from 4 to 1.000 mg/dl (intra-assay coefficient of variation 1.8%); and HDL cholesterol levels ranged from 3 to 120 mg/dl (intra-assay variation coefficient 0.9%). LDL cholesterol was calculated using the Friedewald formula [19]. A standard technique was used to measure the erythrocyte sedimentation rate (ESR) and high-sensitivity C-reactive protein (CRP) level.

Macrophage-specific CEC was measured using boron-dipyrromethene (BODIPY) cholesterol as previously described [8]. Briefly, J774 macrophages were seeded into a 96-well plate at 7 × 10^4^ cells per well. The following day, the cells were incubated for 1 h with BODIPY-tagged cholesterol (25 μM; Avanti Polar Lipids, Alabaster, AL, USA), 0.2% bovine serum albumin (BSA), and 2 μg/ml acetyl-coenzyme A acetyltransferase (ACAT) inhibitor (Sandoz; Sigma-Aldrich, St. Louis, MO, USA) in RPMI 1640 medium plus 1% FBS. Following a washing step with minimal essential medium (MEM)-HEPES, cells were incubated overnight in serum-free RPMI 1640 medium containing 0.3 mM cyclic adenosine monophosphate (cAMP), 0.2% BSA, and 2 μg/ml ACAT inhibitor. Apolipoprotein B-depleted study subject plasma was prepared using polyethylene glycol precipitation. After a washing step with MEM-HEPES, the BODIPY cholesterol-labeled cells were incubated with 2.8% apolipoprotein B-depleted plasma in MEM-HEPES buffer, 0.15 mM cAMP, and 2 μg/ml ACAT inhibitor for 4 h at 37 °C. The resulting quantity of BODIPY cholesterol in the media was measured directly using a spectrofluorometric plate reader (Tecan Trading AG, Männedorf, Switzerland) with an excitation wavelength of 485 nm and emission detection at 530 nm. The CEC was calculated as the amount of BODIPY cholesterol efflux expressed as a fraction of the initial cell content of BODIPY cholesterol. Each assay was performed in triplicate, and when the percentage of variation of every sample was >7%, the sample was reassessed.

### Carotid ultrasound assessment

A carotid ultrasound examination was used to assess CIMT in the common carotid arterial wall and to detect focal plaques in the extracranial carotid tree in patients with RA. A commercially available scanner, the MyLab 70 (Esaote, Genoa, Italy), equipped with a 7- to 12-MHz linear transducer and using an automated software-guided radiofrequency technique called *quality intima-media thickness in real time* was used for this purpose. As previously reported [20], on the basis of the Mannheim consensus, plaque criteria in the accessible extracranial carotid tree (common carotid artery, bulb, and internal carotid artery) were defined as follows: a focal protrusion in the lumen measuring CIMT ≥1.5 mm, a protrusion ≥50% greater than the surrounding CIMT, or arterial lumen encroaching >0.5 mm [21].

### Statistical analysis

In terms of study power, we found a correlation between HDL cholesterol and CIMT (*r* = −0.161, *p* = 0.034). We expected to find a similar relation between CEC and CIMT. To achieve power of 80% to detect differences in the contrast of the null hypothesis, based on assuming a significance bilateral level of 0.05 and by means of analysis of variance in the context of a multiple linear regression model with an expected final determination coefficient of 0.05, it was necessary to include 152 subjects in the study. Demographic and clinical characteristics shown in Table 1 were compared between patients with RA and control subjects using χ^2^ tests for categorical variables or Student’s *t* test for continuous variables (data expressed as mean ± SD). For noncontinuous variables, either the Mann-Whitney *U* test was performed or a logarithmic transformation was performed, and data are expressed as median and IQR. Univariate linear and logistic regression analyses were performed to establish the relationship of demographics, traditional cardiovascular risk factors, lipid profiles, RA-related data, and CEC with both CIMT and the presence of carotid plaque. The relation of CEC with carotid assessments was determined through multivariate linear and logistic regression analysis, adjusting for confounding factors. For the purpose of this study, confounding variables were those with a statistical *p* value <0.20 in the association analysis vis-à-vis both carotid assessment and CEC. For all analyses, we used a 5% two-sided significance level, and all analyses were performed using IBM SPSS Statistics version 21 software (IBM, Armonk, NY, USA) and Stata version 13/SE software (StataCorp, College Station, TX, USA). A *p* value <0.05 was considered statistically significant.

**Table 1:** Characteristics of patients with rheumatoid arthritis and control subjects

	Control subjects (*n* = 223)	Patients with RA (*n* = 178)	*p* Value
Age, years	59 ± 9	55 ± 11	*0.000*
Female sex, *n* (%)	155 (70)	140 (79)	0.063
Body mass index, kg/m^2^	28 ± 5	28 ± 5	0.74
Abdominal circumference, cm	93 ± 14	97 ± 13	*0.006*
Systolic blood pressure, mmHg	133 ± 15	137 ± 19	*0.018*
Diastolic blood pressure, mmHg	82 ± 10	83 ± 12	0.36
Cardiovascular comorbidity			
Smoking, *n* (%)	45 (20)	29 (16)	0.29
Diabetes, *n* (%)	10 (4)	27 (15)	*0.000*
Hypertension, *n* (%)	64 (29)	62 (35)	0.22
Dyslipidemia, *n* (%)	41 (18)	71 (40)	*0.000*
Antihypertensive treatment, *n* (%)	38 (17)	63 (35)	*0.000*
Statins, *n* (%)	22 (10)	60 (34)	*0.000*
Hormone replacement therapy, *n* (%)	7 (3)	0 (0)	*0.018*
Laboratory examinations, including lipid profile			
ESR, mm/h	10 ± 8	35 ± 22	*0.000*
CRP, mg/dl	1.0 (1.0–3.0)	3.3 (1.6–6.1)	0.34
Cholesterol, mg/dl	218 ± 39	206 ± 37	*0.001*
Triglycerides, mg/dl	105 ± 52	151 ± 92	*0.000*
HDL cholesterol, mg/dl	63 ± 17	56 ± 16	*0.000*
LDL cholesterol, mg/dl	134 ± 36	120 ± 33	*0.000*
Lipoprotein A, mg/dl	16 (9–35)	33 (10–121)	*0.000*
Apolipoprotein A, mg/dl	191 ± 35	170 ± 28	*0.000*
Apolipoprotein B, mg/dl	102 ± 24	109 ± 59	0.13
ApoB/ApoA ratio	0.55 ± 0.16	0.65 ± 0.29	*0.000*
Atherogenic index	3.72 ± 1.14	4.02 ± 1.51	*0.036*
Cholesterol efflux capacity, %	16.9 ± 10.4	18.9 ± 9.0	0.11
Rheumatoid arthritis-related data			
Disease duration, years		7 (4–15)	
Age at onset, years		45 ± 13	
DAS28		3.74 ± 1.19	
Remission, *n* (%)		38 (21)	
Low activity, *n* (%)		29 (16)	
Moderate activity, *n* (%)		84 (47)	
High activity, *n* (%)		27 (15)	
DAS28-CRP		2.94 ± 0.99	
SDAI		14 (8–21)	
CDAI		82 (40–112)	
Rheumatoid factor, *n* (%)		119 (67)	
ACPA, *n* (%)		98 (55)	
Prednisone intake, *n* (%)		62 (35)	
Prednisone dose, mg/day		5 (3–6)	
NSAIDs, *n* (%)		78 (44)	
DMARDs, *n* (%)		153 (86)	
Methotrexate, *n* (%)		135 (76)	
Leflunomide, *n* (%)		19 (11)	
Biologic therapy, *n* (%)		41 (23)	
Anti-TNF-α therapy, *n* (%)		23 (13)	
Tocilizumab, *n* (%)		11 (6)	
Rituximab, *n* (%)		5 (3)	
Abatacept, *n* (%)		2 (1)	
Carotid assessments			
CIMT, mm		0.671 ± 0.143	
Carotid plaque, *n* (%)		66 (37)	

Data represent mean (SD) or median (IQR) when data were not normally distributed

*Abbreviations: CRP* C-reactive protein, *LDL* Low-density lipoprotein, *NSAID* Nonsteroidal anti-inflammatory drug; *DMARD* Disease-modifying antirheumatic drug, *ESR* Erythrocyte sedimentation rate, *DAS28* Disease Activity Score in 28 joints, *HDL* High-density lipoprotein, *SDAI* Simplified Disease Activity Index, *CDAI* Clinical Disease Activity Index, *TNF-*α Tumor necrosis factor-α, *ACPA* Anticitrullinated protein antibody, *CIMT* Carotid intima-media thickness. Significant '*p*' values are higlighted in italics

## Results

### Demographic, laboratory, and disease-related data

A total of 401 sex-matched participants, 178 patients with RA, and 223 control subjects were included in this study. Demographic and disease-related characteristics of the participants are shown in Table 1. There were no differences between patients and control subjects with regard to body mass index. However, abdominal circumference and the presence of hypertension, dyslipidemia, or diabetes were more common in patients with RA. Similarly, statin intake was more frequently observed in patients with RA than in control subjects (34% versus 10%, *p* = 0.000). Patients with RA had moderately active disease, as shown by the mean DAS28 (3.74 ± 1.19). More than one-third (35%) were taking prednisone (median dose of the 62 patients on prednisone was 5 [IQR 3–6] mg/day at the time of the study). As expected, ESR values were statistically significantly higher in patients than in control subjects. One hundred nineteen (67%) patients had positive test results for rheumatoid factor, 153 (86%) were taking disease-modifying antirheumatic drugs, and 23 (13%) were receiving anti-TNF-α therapy.

The mean CEC of HDL was not significantly different between patients with RA (18.9 ± 9.0%) and control subjects (16.9 ± 10.4%) (*p* = 0.11). This difference remained nonsignificant after adjusting for HDL cholesterol levels, age, sex, and statin use (data not shown).

### Relationship of CEC with demographic, traditional cardiovascular risk factors, and disease-related data in patients and control subjects

Demographic variables were not associated with CEC, except for a correlation with male sex that was found only in patients with RA and not in control subjects. Systolic blood pressure was inversely correlated with CEC in control subjects (β coefficient −0.1 [−0.2 to 0.0]%, *p* = 0.025). In patients with RA, a similar trend was found, although a statistically significant difference was not reached. Neither the traditional cardiovascular risk factors nor the cardiovascular comorbidity-related data were associated with CEC. Similarly, the lipid profile did not show any relationship with CEC in patients or control subjects (Table 2).

**Table 2:** Univariate relationship of traditional cardiovascular risk factors and rheumatoid arthritis-related data with high-density lipoprotein cholesterol efflux capacity in control subjects and patients with rheumatoid arthritis

	Control subjects		Patients with RA	
	Percent efflux β coefficient (95% CI)	*p* Value	Percent efflux β coefficient (95% CI)	*p* Value
Age, years	−0.9 (−0.4 to 0.2)	0.50	−0.2 (−0.2 to 0.1)	0.78
Male sex	−0.9 (−4.6 to 2.8)	0.62	−6.1 (−9.4 to 2.8)	*0.000*
Body mass index	−0.2 (−0.6 to 0.1)	0.20	0.2 (−0.1 to 0.5)	0.21
Abdominal circumference	−0.1 (−0.2 to 0.0)	0.058	0.0 (−0.1 to 0.2)	0.42
Systolic blood pressure	**−0.1 (−0.2 to 0.0)**	*0.025*	0.1 (−0.0 to 0.2)	0.11
Diastolic blood pressure	−0.0 (-0.1 to 0.0)	0.29	0.01 (−0.0 to 0.2)	0.15
Cardiovascular comorbidities				
Smoking	1.8 (−2.4 to 6.0)	0.40	−0.3 (−4.5 to 3.9)	0.89
Diabetes	1.2 (−5.9 to 8.3)	0.74	2.9 (−1.3 to 7.2)	0.17
Hypertension	−2.4 (−6.0 to 1.1)	0.17	0.1 (−3.0 to 3.2)	0.95
Dyslipidemia	−1.1 (−5.2 to 3.1)	0.61	−0.3 (−3.4 to 2.8)	0.85
Antihypertensive treatment	0.7 (−3.6 to 5.0)	0.75	0.4 (−2.7 to 3.5)	0.80
Statins	−2.1 (−7.4 to 3.2)	0.44	0.6 (−2.6 to 3.8)	0.70
Laboratory examinations, including lipid profile				
ESR	−0.2 (−0.1 to 0.1)	0.23	−0.1 (−0.1 to 0.0)	0.12
CRP	−0.3 (−0.6 to 0.0)	0.092	−0.0 (−0.1 to 0.1)	0.83
Cholesterol	0.0 (−0.0 to 0.1)	0.11	0.00 (−0.0 to 0.00)	0.69
Triglycerides	−0.0 (−0.1 to 0.0)	0.19	0.0 (−0.0 to 0.0)	0.30
HDL cholesterol	0.1 (−0.0 to 0.2)	0.092	0.0 (−0.1 to 0.1)	0.48
LDL cholesterol	0.0 (−0.0 to 0.1)	0.32	−0.0 (−0.1 to 0.0)	0.62
Lipoprotein A	0.1 (−0.0 to 0.1)	0.059	−0.0 (−0.0 to 0.0)	0.35
Apolipoprotein A	0.0 (−0.0 to 0.1)	0.089	−0.0 (−0.1 to 0.0)	0.84
Apolipoprotein B	0.0 (−0.1 to 0.1)	0.77	0.00 (−0.0 to 0.0)	0.99
ApoB/ApoA ratio	−5.8 (−16.8 to 5.4)	0.31	0.8 (−4.1 to 5.7)	0.76
Atherogenic index	0.7 (−2.4 to 1.0)	0.41	0.0 (−1 to 1)	0.99
Rheumatoid arthritis-related data				
Disease duration			0.1 (−0.0 to 0.3)	0.12
DAS28			−1.1 (−2.3 to 0.2)	0.086
Remission			–	
Low disease activity			**−5.2 (−10.0 to 0.3)**	*0.039*
Moderate disease activity			**−4.6 (−8.5 to 0.7)**	*0.020*
High disease activity			−3.2 (−8.1 to 1.7)	0.19
Moderate and high disease activity			−**4.2 (−7.9 to 0.6)**	*0.024*
DAS28-CRP			−1.1 (−2.7 to 0.4)	0.14
SDAI			−0.0 (−0.1 to 0.1)	0.54
CDAI			−0.0 (−0.0 to 0.0)	0.31
Rheumatoid factor			−0.4 (−3.8 to 2.9)	0.81
ACPA			0.4 (−2.7 to 3.6)	0.78
Prednisone intake			−0.3 (−3.4 to 2.9)	0.88
Prednisone dose			−0.3 (−3.4 to 2.9)	0.88
NSAIDs			**4.6 (1.5 to 7.6)**	*0.004*
DMARDs			−0.2 (−4.6 to 4.2)	0.93
Methotrexate			0.5 (−2.9 to 4.0)	0.77
Leflunomide			−1.3 (−5.9 to 3.3)	0.58
Biologic therapy			3.57 (−0.0 to 7.2)	0.052
Anti-TNF-α therapy			0.7 (−4.0 to 5.5)	0.76
Tocilizumab			**8.0 (2.3 to 13.7)**	*0.007*
Rituximab			1.3 (−9.1 to 11.7)	0.81
Abatacept			−1.4 (−14.1 to 11.4)	0.83

DAS28 relation with cholesterol efflux capability was studied using remission category as the reference category

*Abbreviations: CRP* C-reactive protein, *LDL* Low-density lipoprotein, *NSAID* Nonsteroidal anti-inflammatory drug; *DMARD* Disease-modifying antirheumatic drug, *ESR* Erythrocyte sedimentation rate, *DAS28* Disease Activity Score in 28 joints, *HDL* High-density lipoprotein, *SDAI* Simplified Disease Activity Index, *CDAI* Clinical Disease Activity Index, *TNF-*α Tumor necrosis factor-α, *ACPA* Anticitrullinated protein antibody, *CIMT* Carotid intima-media thickness, *ApoA* Apolipoprotein A, *ApoB* Apolipoprotein B. Significant '*p*' values are higlighted in italics

Disease activity, when considered continuous, was not associated with lower levels of CEC (β coefficient −1.1 [−2.3 to 0.2]%, *p* = 0.086). Nevertheless, when patients were stratified according to the degree of disease activity, some differences were seen. With respect to this, the patient subgroup that exhibited low (β coefficient −5.2 [−10.0 to 0.3]%, *p* = 0.039) and moderate (β coefficient −4.6 [−8.5 to 0.7]%, *p* = 0.020) disease activity was associated with statistically significant lower levels of CEC than those in clinical remission. Although no association was found when patients with RA with high disease activity were compared with those in remission (β coefficient −3.2 [−8.1 to 1.7]%, *p* = 0.19), this association was maintained when moderate and high disease activity groups were considered as a single group (β coefficient −4.2 [−7.9 to 0.6]%, *p* = 0.024).

Neither rheumatoid factor- nor anticitrullinated protein antibody (ACPA)-positive status was associated with CEC. Apart from those patients who underwent tocilizumab treatment (11 patients), in whom the use of this anti-interleukin-6 biologic agent led to higher CEC levels (β coefficient 8.0 [2.3–13.7]%, *p* = 0.007), as well as a correlation linking nonsteroidal anti-inflammatory drug (NSAID) intake and a higher CEC index (β coefficient 4.6 [1.5–7.6]%, *p* = 0.004), no association between RA therapy and CEC was found (Table 2).

### Relationship of RA patient characteristics with CIMT and carotid plaques

Age, male sex, waist circumference, and traditional cardiovascular risk factors (hypertension, diabetes, and dyslipidemia), except for smoking, positively correlated with either the presence of carotid plaque or CIMT. With respect to laboratory data, lipoprotein A (OR 1.00 [95% CI 1.00–1.01], *p* = 0.043) was associated with the presence of carotid plaque. Whereas triglycerides (*p* = 0.034) and atherogenic index (*p* = 0.023) correlated with higher levels of CIMT, HDL cholesterol showed a negative association with CIMT (β coefficient −0.01 [−0.03 to 0.00], *p* = 0.034). Levels of ESR were positively associated with both carotid plaque and CIMT.

Regarding RA-related data, disease activity as assessed by DAS28 (OR 1.36 [95% CI 1.05–1.77], *p* = 0.022) or CDAI (OR 1.01 [95% CI 1.00–1.01], *p* = 0.055) was associated with a higher risk of carotid plaque involvement. Rheumatoid factor was negatively and marginally related to a lower level of CIMT, though no association of carotid plaque or CIMT with ACPA status was found. NSAID intake was negatively associated with both carotid plaque (OR 0.40 [95% CI 0.21–0.76], *p* = 0.005) and CIMT (β coefficient −0.6 [−1.0 to 0.2], *p* = 0.005) (Table 3). However, no association between prednisone intake or prednisone dose and carotid plaque or CIMT was found (Table 3).

**Table 3:** Univariate relationship of characteristics of patients with rheumatoid arthritis with carotid intima-media thickness and carotid plaques

	Carotid plaque		CIMT (×10 mm)	
Patients with RA (*n* = 178)	OR (95% CI)	*p* Value	β Coefficient (95% CI)	*p* Value
Age	1.15 (1.10–1.20)	*0.000*	0.08 (0.01–0.01)	*0.000*
Male sex	3.00 (1.44–6.26)	*0.003*	1.1 (0.6–1.6)	*0.000*
Body mass index	1.00 (0.95–1.06)	0.92	0.03 (0.02–0.07)	0.23
Abdominal circumference	1.01 (0.99–1.04)	0.31	0.02 (0.00–0.03)	**0.021**
Systolic blood pressure	1.04 (1.02–1.06)	*0.000*	0.04 (0.03–0.05)	*0.000*
Diastolic blood pressure	1.02 (0.997–1.05)	0.078	0.02 (0.01–0.04)	*0.009*
Cardiovascular comorbidity				
Smoking	1.04 (0.46–2.37)	0.92	−0.1 (0.7 to 0.4)	0.63
Diabetes	3.54 (1.51–8.30)	*0.004*	1.0 (0.04 to 1.6)	*0.001*
Hypertension	2.57 (1.36–4.87)	*0.004*	0.6 (0.2 to 1.1)	*0.005*
Dyslipidemia	3.61 (1.91–6.84)	*0.000*	0.4 (−0.01 to 0.8)	0.082
Antihypertensive treatment	2.73 (1.44–5.18)	*0.002*	0.7 (0.2 to 1.1)	*0.002*
Statins	3.93 (2.04–7.58)	*0.000*	0.5 (0.0 to 0.9)	*0.036*
Laboratory including lipid profile				
ESR	1.02 (1.01–1.04)	*0.003*	0.01 (0.00 to 0.02)	*0.041*
CRP	0.45 (0.96–1.02)	0.45	0.00 (−0.01 to 0.01)	0.947
Cholesterol	1.00 (0.99–1.01)	0.83	0.00 (0.00 to 0.01)	0.16
Triglycerides	1.00 (0.99–1.01)	0.13	0.00 (0.00 to 0.00)	*0.034*
HDL cholesterol	0.99 (0.98–1.01)	0.51	−0.01 (−0.03 to 0.00)	*0.034*
LDL cholesterol	0.99 (0.99–1.01)	0.77	0.00 (0.00 to 0.01)	0.14
Lipoprotein A	1.00 (1.00–1.01)	*0.043*	0.00 (0.00 to 0.00)	0.76
Apolipoprotein A	1.01 (0.99–1.02)	0.37	0.00 (−0.01 to 0.01)	0.80
Apolipoprotein B	0.99 (0.99–1.01)	0.73	0.00 (0.00 to 0.00)	0.48
ApoB/ApoA ratio	0.70 (0.21–2.33)	0.56	0.4 (0.4 to 1.1)	0.36
Atherogenic index	1.06 (0.87–1.29)	0.57	0.2 (0.0 to 0.3)	*0.029*
Rheumatoid arthritis-related data				
Disease duration	1.02 (0.99–1.06)	0.17	0.01 (−0.01 to 0.03)	0.41
DAS28	1.36 (1.05–1.77)	*0.022*	0.12 (−0.06 to 0.30)	0.18
DAS28-CRP	1.19 (0.88–1.63)	0.26	0.08 (−0.14 to 0.29)	0.47
SDAI	0.99 (0.98–1.02)	0.85	0.00 (−0.01 to 0.01)	0.76
CDAI	1.01 (1.00–1.01)	*0.055*	0.00 (0.00 to 0.01)	0.16
Rheumatoid factor	0.87 (0.45–1.71)	0.69	−0.5 (−1.0 to 0.0)	*0.043*
ACPA	0.87 (0.46–1.63)	0.66	−0.3 (−0.8 to 0.1)	0.14
Prednisone intake	1.11 (0.59–2.10)	0.74	0.0 (−0.4 to 0.5)	0.90
Prednisone doses	0.99 (0.89–1.09)	0.81	−0.04 (−0.1 to 0.2)	0.20
NSAIDs	0.40 (0.21–0.76)	*0.005*	−0.6 (−1.0 to 0.2)	*0.005*
DMARDs	2.04 (0.77–5.41)	0.15	0.3 (−0.4 to 0.9)	0.43
Methotrexate	0.77 (0.38–1.54)	0.46	−0.3 (−0.8 to 0.2)	0.25
Leflunomide	2.04 (0.78–5.32)	0.14	0.9 (0.2 to 1.6)	*0.010*
Biologic therapy	0.51 (0.23–1.13)	0.098	−0.2 (−0.8 to 0.3)	0.35
Anti-TNF-α therapy	0.89 (0.36–2.23)	0.81	0.4 (−0.3 to 1.0)	0.23
Tocilizumab	0.16 (0.02–1.26)	0.081	−0.7 (−1.6 to 0.1)	0.085
Rituximab	0.42 (0.05–3.80)	0.44	−1.1 (−2.4 to 0.1)	0.81
Abatacept	1.71 (0.11–27.8)	0.71	0.6 (−1.4 to 2.6)	0.55

*Abbreviations: CRP* C-reactive protein, *LDL* Low-density lipoprotein, *NSAID* Nonsteroidal anti-inflammatory drug; *DMARD* Disease-modifying antirheumatic drug, *ESR* Erythrocyte sedimentation rate, *DAS28* Disease Activity Score in 28 joints, *HDL* High-density lipoprotein, *SDAI* Simplified Disease Activity Index, *CDAI* Clinical Disease Activity Index, *TNF-*α Tumor necrosis factor-α, *ACPA* Anticitrullinated protein antibody, *CIMT* Carotid intima-media thickness, *ApoA* Apolipoprotein A, *ApoB* Apolipoprotein B. Significant '*p*' values are higlighted in italics

### CEC’s association with carotid subclinical atherosclerosis in patients with RA

Higher CEC was associated with a protective effect against the presence of carotid plaque in patients with RA. This association (OR 0.94 [95% CI 0.89–0.98], *p* = 0.015) was maintained even after multivariate analysis (adjusted for age, sex, systolic blood pressure, diabetes, ESR, disease duration, DAS28, and tocilizumab use). In contrast, CEC was not found to be associated with CIMT in patients with RA (Table 4).

**Table 4:** Cholesterol efflux capacity relationship with carotid intima-media thickness and carotid plaque

	Carotid plaque		CIMT (×10 mm)	
Cholesterol efflux capacity	OR (95% CI)	*p*	β Coefficient (95% CI)	*p*
Unadjusted	**0.95 (0.92–0.99)**	**0.023**	0.01 (−0.02 to 0.03)	0.67
Adjusted	**0.94 (0.89–0.98)**	**0.015**	0.01 (−0.02 to 0.03)	0.54

Adjusted for age, sex, systolic blood pressure, diabetes, ESR, disease duration, DAS28 and tocilizumab use

*CIMT* Carotid intima-media thickness

## Discussion

In the present study, we show, for the first time to our knowledge, that CEC is independently associated with carotid plaque in patients with RA. Additionally, CEC was shown to be inversely proportional to disease activity, with CEC being lower in patients with low or moderate activity than in patients in remission.

In our study, we did not observe a difference in CEC between patients and control subjects, a finding that is in agreement with previous reports. In fact, using an assay similar to that of our own study, Charles-Schoeman et al*.* found no significant difference between 40 patients with RA and 40 age- and sex-matched healthy control subjects [9]. Ronda et al*.* [10] studied CEC through 4 different and specifically CEC pathways in 30 patients with RA and 30 healthy control subjects. They did not discover any significant differences in scavenger receptor class B member 1 (SR-BI)-mediated efflux, ATP-binding cassette A1 (ABCA1)-mediated efflux, and aqueous diffusion (AD) CEC pathways. Only ATP-binding cassette G1 (ABCG1)-mediated efflux was found to be impaired when patients with RA were compared with healthy control subjects.

Regarding the relationship of disease activity with CEC, our findings are also in agreement with other previous reports [9, 10]. In our study, DAS28, on a continuous basis, showed a trend toward being inversely related with CEC. Interestingly, patients with low and moderate disease activity had statistically significant lower CEC than those in remission. However, CEC was not significantly different in patients with high disease activity when compared with those in remission. A lack of statistical power when comparing the high disease activity group with patients in remission may be the reason for this result because low and moderate disease activity levels were linked to lower levels of CEC. Additionally, when patients with moderate and high disease activity were included in a single group and compared with those in remission, the statistically significant association was maintained. For this reason, we believe that the association between disease activity and CEC found in our study is robust enough to be considered real. In keeping with our findings, Charles-Schoeman et al. [9] and Ronda et al. [10] both described a relationship between disease activity and CEC in their studies. In the former, significant differences were noted between patients with RA with low disease activity/clinical remission and patients with RA with high disease activity [9]. Significant correlations were also found between CEC and RA disease activity and systemic inflammation as measured by ESR [9]. In the latter study [10], a significant inverse correlation was found between ABCG1-mediated CEC values and DAS28 in patients with RA.

No other RA-related data different from the disease activity findings were found to be associated with CEC in our study. Only tocilizumab and NSAIDs showed a relationship with CEC. The fact that tocilizumab treatment was associated with increased levels of CEC is in agreement with two recent reports that described a beneficial effect of tocilizumab on CEC over time [22, 23]. However, the association of tocilizumab found in our study should be interpreted with caution, given the small number of patients included in our series. In our study, NSAIDs were also associated with a protective effect for CIMT and carotid plaque, as well as with higher levels of CEC. This is in agreement with previous reports supporting the notion that in patients with inflammatory arthritis, the anti-inflammatory effect of NSAIDs may compensate for the potentially increased risk of cardiovascular disease associated with these drugs [24, 25]. To the best of our knowledge, the beneficial effects of NSAIDs over CEC have not previously been described. However, we did not find an association of prednisone intake with carotid subclinical atherosclerosis. On one hand, we believe that this is due to the fact that the risk of corticosteroids over cardiovascular disease is dose-dependent and may be lower or absent in patients receiving low-dose glucocorticoid therapy [26]. On the other hand, we do not have an explanation for the marginal protective effect of rheumatoid factor over CIMT found in our series of white patients with RA. It could be the result of a spurious correlation that needs to be replicated in further studies. Nevertheless, a recent study has shown an association between RA-related autoantibodies with subclinical and clinical atherosclerosis in African American women but not in white women or men [27].

The absence of any association between traditional cardiovascular risk factors or lipid profile with CEC in both patients and control subjects is in agreement with previous reports. In this sense, traditional risk factors reportedly explain only 3% of the variance observed in CEC [8]. Moreover, glucose tolerance status does not appear to impact CEC [28], and CEC cannot be explained by HDL cholesterol or apolipoprotein A-I levels [29]. Similarly, we did not find any association of statins with CEC in patients and control subjects. This finding supports the claim that statins most likely exert therapeutic benefit by means of a mechanism that is different from the promotion of cholesterol efflux [7]. Smoking has also been found to be a significant inverse predictor of CEC in previous studies [7]. However, we did not find this association in our study. On one hand, we think it could be due to our study design because we included only current smokers. On the other hand, only 16% of the patients with RA included in our series were current smokers at the time of the assessment. Nevertheless, we feel that CEC impairment in RA may be predominantly the result of an inflammation-related disturbance rather the effect of traditional cardiovascular risk factors.

CEC was associated with carotid plaque in our study. However, this was not the case for CIMT. We believe that the relationship of CEC with CIMT is probably not linear; thus, linear regression may have failed to detect this association. Nevertheless, carotid plaque is considered to be a better predictor of cardiovascular disease than CIMT [30]. It is also known that whereas plaque reflects advanced atherosclerosis and associates closely with dyslipidemia, increased CIMT represents mostly high blood pressure-mediated arterial medial hypertrophy and relates more strongly to left ventricular hypertrophy and stroke [31]. For this reason, we think that the association with carotid plaque and not with CIMT found in our study is consistent with previous knowledge regarding the etiopathogenesis of atherosclerotic disease.

We acknowledge several limitations in our study. First, carotid assessments were not available for healthy control subjects. Although CEC has been widely associated with CIMT and cardiovascular events in the general population, the availability of carotid assessments in control subjects would have allowed us to study a different effect or statistical interaction between these two populations. Second, as previously mentioned, CEC pathways are diverse, and some other molecules may be implicated in CEC. Finally, although there are other ways of assessing cholesterol efflux in vitro, most research done in population-based cohorts has been carried out using the same assay as the one described in our study. This assay integrates the pathways known to mediate cholesterol efflux from macrophages (i.e., ABCA1, ABCG1, SR-BI, and AD).

## Conclusions

Our study, which includes the largest series of patients with RA ever assessed for CEC, reveals for the first time, to our knowledge, that CEC is related to subclinical atherosclerosis in patients with RA. The fact that CEC is also associated with disease activity reinforces the idea that CEC may be a mediator between disease activity and subclinical atherosclerosis. We emphasize the potential role of checking CEC in patients with RA because it may be a complementary approach to the assessment of atherosclerotic disease in these patients. We feel that our findings herald a new opportunity for research in this area in which future investigations are warranted.

## Abbreviations

ABCA1: ATP-binding cassette A1
ABCG1: ATP-binding cassette G1
ACAT: Acetyl-coenzyme A acetyltransferase
ACPA: Anticitrullinated protein antibody
AD: Aqueous diffusion
ApoA: Apolipoprotein A
ApoB: Apolipoprotein B
BODIPY: Boron-dipyrromethene
BSA: Bovine serum albumin
cAMP: Cyclic adenosine monophosphate
CDAI: Clinical Disease Activity Index
CEC: Cholesterol efflux capacity
CRP: C-reactive protein
CIMT: Carotid intima-media thickness
DAS28: Disease Activity Score in 28 joints
DMARD: Disease-modifying antirheumatic drug
ESR: Erythrocyte sedimentation rate
HDL: High-density lipoprotein
LDL: Low-density lipoprotein
MEM: Minimal essential medium
NSAID: Nonsteroidal anti-inflammatory drug
RA: Rheumatoid arthritis
SDAI: Simplified Disease Activity Index
SR-B1: Scavenger receptor class B member 1
TNF-α: Tumor necrosis factor-α
